# Supplementary material for: A Qualitative Study of Rural Plant-Based Eaters’ Knowledge and Practices for Nutritional Adequacy
Source: Nutrients. 2024 Oct 16;16(20):3504. doi: 10.3390/nu16203504 (PMC11510633; doi:10.3390/nu16203504)
Supplement: Supplementary file 1 [file nutrients-16-03504-s001.zip › Supplement S1. Diet Survey.pdf]

Supplement A. Demographic and Diet Survey

Are you aged 18 years or older?

○ Yes

○ No

What is your zip code?

____

Have you lived in a rural area for at least two years?

○ Yes

○ No

In the typical week, how many times do you eat red meat, poultry or fish?

○ Five times or more

○ Four times or fewer

○ I don't eat any red meat, poultry or fish

In the typical day, how many cups of vegetables do you eat? (Examples of 1 cup of vegetables include 3 broccoli spears, 1 cup of cooked leafy greens, 2 cups of lettuce, 12 baby carrots, 1 large sweet potato, or 1 large tomato)

○ 2.5 cups or more

○ Less than 2.5 cups

○ I do not eat any vegetables

In the typical day, how many cups of fruit do you eat? (Examples of 1 cup of fruit include 1 small apple, 1 large banana, 1 large orange, 8 large strawberries, 1 medium pear, 2 large plums, or 1/2 cup of dried fruit.)

- 2 cups or more
- Less than 2 cups
- I do not eat any fruit

In general, do you believe that you are a healthy eater?

- Yes
- No

Are you willing to take part in an audio-recorded interview?

- Yes
- No

Do you share a household with anyone who has previously participated in this study?

- Yes
- No

In what county do you live?
____

Which if the following dietary patterns do you most closely identify with?

- ○ Pesco-vegetarian (consuming no meat but fish)
- ○ Flexitarian (limiting consumption of red meat, poultry or fish to 4 or fewer times per week)
- ○ Vegan (not consuming any animal products)
- ○ Vegetarian (not consuming any red meat, poultry or fish)
- ○ Other ____

Compared to the average American, I would rate my nutrition knowledge as:

- ○ Above Average
- ○ Average
- ○ Below Average

Are you concerned about adequacy of any of the following nutrients in your diet? Select all that apply:

- ☐ Protein
- ☐ Omega-3 fatty acids
- ☐ Iron
- ☐ Zinc
- ☐ Iodine
- ☐ Calcium
- ☐ Vitamin D

- [ ] Vitamin B-12
- [ ] Other (specify) ____
- [ ] None

Do you regularly take any of the vitamin or mineral supplements listed below? Select all that apply:

- [ ] Multi-vitamin
- [ ] Omega-3 fatty acids
- [ ] Iron
- [ ] Zinc
- [ ] Iodine
- [ ] Calcium
- [ ] Vitamin D
- [ ] Vitamin B-12
- [ ] Other (specify) ____
- [ ] None

How often do you consume the following foods or dietary supplements?

	Never/Almost Never (never or very rarely)	Sometimes (less than once a week)	Often (more than once a week)
Nutritional Yeast	○	○	○
Walnuts	○	○	○
Chia Seeds	○	○	○
Flax Seeds	○	○	○
Hemp Seeds	○	○	○
Algae/Spirulina	○	○	○
Fortified non-dairy milks	○	○	○
Seaweed	○	○	○
Iodized Salt	○	○	○
Protein Powder	○	○	○

With what gender do you identify?

- ○ Female
- ○ Male
- ○ Non-binary/third gender
- ○ Prefer to self-describe ____
- ○ Prefer not to answer

In what year were you born?

____

hispanic Are you of Hispanic, Latino, or Spanish Origin?

- ○ No, I am not of Hispanic, Latino or Spanish Origin
- ○ Yes, I am of Hispanic, Latino or Spanish Origin

race What is your race? Select all that apply

- ☐ American Indian or Alaskan Native
- ☐ Black or African American
- ☐ Asian
- ☐ Native Hawaiian or Other Pacific Islander
- ☐ White
- ☐ Other race or origin ____

What is the highest level of formal education that you have completed?

- ◯ Some high school (no diploma)
- ◯ High school graduate (including GED)
- ◯ Some college (no degree)
- ◯ Associates degree/technical school/apprenticeship
- ◯ Bachelor’s degree
- ◯ Postgraduate (e.g. Masters, PhD), / progressional degree (e.g. JD)

Which of the following best describes your household income range in 2021 before taxes? Household income includes people currently living within your home, including family and non-family members.

- ◯ Less than $24,999 per year
- ◯ $25,000-$74,999 per year
- ◯ $75,000-$99,999 per year
- ◯ More than $100,000 per year

Please fill out the following information so that we can contact you for your interview and provide the gift card upon completion.

- ◯ Phone ____
- ◯ Email ____
- ◯ Street Address ____
- ◯ City ____
- ◯ State ____

Postal code ____
