# Supplementary material for: A Qualitative Study of Rural Plant-Based Eaters’ Knowledge and Practices for Nutritional Adequacy
Source: Nutrients. 2024 Oct 16;16(20):3504. doi: 10.3390/nu16203504 (PMC11510633; doi:10.3390/nu16203504)
Supplement: Supplementary file 1 [file nutrients-16-03504-s001.zip › Supplement S2. Semi-Structured Interview Guide.pdf]

Supplement 2. Semi-Structured Interview Guide.

- **Opening Questions that Develop Rapport**:
    - Can you tell me about what you ate for dinner last night?
        - Probe: Is this a "typical" meal for you?
        - Probe: If not, what would be considered typical?

    - Overall, how would you describe the way that you eat?
        - Probe: If you were to tell a friend about the types of foods you eat, how you identify, those sorts of things.

- **SHOW DIAGRAM OF A PLANT-BASED DIET (Image 1)**
    - Define plant-based diet in the context of this study
        - A dietary pattern **dominated by fresh fruits and vegetables and minimally processed plant-foods** and *decreased* consumption of meat and animal products. It doesn't necessarily mean vegan or vegetarian, but it includes them. You'll see in this diagram, that about half the plate is fruit and vegetables, and we've got these smaller slivers for meat and animal products (or if someone wanted to eat plant-base alternatives), and here this section for plant sourced proteins, like nuts seeds and legumes, this section would get bigger, as someone eats less animal-based proteins from the other section.

- **Personal Perspectives and Experiences of Eating Plant-Based**:
    - I'm curious to learn more about why you eat a plant-based dietary pattern.
        - Probe: What are some of the reasons that you do it?

    - What was your experience starting a plant-based diet?
        - Probe: How long have you been eating this way?

    - On a 10-point scale from 'very easy' to 'very difficult', how would you categorize your personal experience eating a plant-based diet in rural Vermont?
        - Probe: What made you choose this number?
        - Probe: What factors make it easy?
        - Probe: What factors make it difficult? (cost, time, availability, seasonality, etc?)

- **Outside Perceptions of Plant-Based Diet:**
    - What do you think the people that you normally eat with think about a plant-based diet?
        - Probe: Do you ever get pushback?
        - Probe: Do the people around you eat in similar ways?
        - Probe: Are there differences between friends and family?
        - Probe: What do you siblings think?

    - What do you think other people in your community think about plant-based diets?

- **Community Recommendations**:
    - If you were to give tips to a friend or a neighbor about how to eat a plant-based diet, what would you say?

        - Probe: Do you have tips, hacks or recommendations that make eating a plant-based diet easier in general?
        - Probe: How did you learn about that?

    - If you could make a change in your community that would make it easier for other people to eat plant-based, what would it be?
        - Probe: Tell me more about that.
        - Probe: How would you suggest that change gets implemented?

- **Questions about nutrients of concern**:
    - Do you have any concerns about the overall healthfulness of a plant-based diet?
        - Probe: Tell me about them.
        - Probe: Are there any nutrients that you are concerned about on a plant-based diet?

    - What do you think about when choosing plant-based protein foods?
        - Probe: What are your thoughts on plant-based meat alternatives?

    - Do you seek out any specific foods or food combinations to fill in possible gaps in your diet from eating very little animal products?
        - For example: Peanut butter and jelly sandwich to make a complete protein or nutritional yeast for B12?
        - Probe: I am interested to hear more about that.

    - ***Specific questions based on responses to the survey such as:***
        - Nutrients Concerned About:
            - I see from the survey that you are not concerned about any nutrients; can you tell me more about what you do to cover all of your nutritional bases?
            - I also noticed that you put ______ as a nutrient that you're concerned about, and I'm curious to see if you've ever thought about taking a supplement for that as well?
            - I see that you are concerned about ____ can you tell me a little bit about that?
        - Vitamins/Supplements Regularly Taken:
            - From the survey I can see that you take a few supplements like ______. Can you tell me a little but more about your decision to take them? How long have you been taking them?
            - From the survey I see that you don’t take any supplements, can you tell me a little bit more about that?
        - Foods Consumed Often:
            - From the survey, I see that that _____ are a staple in your diet- can you tell me a little bit more about how those fit into your diet?
        - Foods Consumed Rarely/Never:
            - I see that you rarely or never eat foods like ______. What comes to mind when you think about those foods?

- **Closing questions**:
    - Is there anything else about eating a plant-based diet in your community that you would like to share?
    - Is there Anything else about diet planning that we haven't talked about that you would like to mention?
