# Supplementary material for: A Qualitative Study of Rural Plant-Based Eaters’ Knowledge and Practices for Nutritional Adequacy
Source: Nutrients. 2024 Oct 16;16(20):3504. doi: 10.3390/nu16203504 (PMC11510633; doi:10.3390/nu16203504)
Supplement: Supplementary file 1 [file nutrients-16-03504-s001.zip › Supplement S3. Nutrients of Concern Codebook.pdf]

Supplement 3. Nutrients of Concern Codebook.

<u>CODING GUIDELINES</u>

- Anytime a **<u>primary</u>** question directly precedes a piece of text to be coded, include the question in the coded text. However, if the question and the relevant text are separated, there is no need to code the question.
- Include **<u>follow-up</u>** questions immediately preceding a piece of text only if it helps to clarify the content.
- If there is back and forth in an exchange, do not code the middle content if it is not relevant; make it two separate sections as opposed to one large one with non-relevant information in the middle.
- Only code as much text as is relevant to clarify meaning and context of a comment.
- Include interviewer/interviewee identifiers
- Do not include "ums" etc., unless they occur in the middle of a segment of text.
- Code complete sentences.

Code	Definition	When to Use	When Not to Use	Examples
**AA. NUTRIENTS OF CONCERN AND SUPPLEMENTATIO N**	*Discussion regarding specific nutrients of concern in a plant-based diet in either the context of general nutrition or supplementation*			
**AA1.** OMEGA-3 FATTY ACIDS	See AA.	See AA.	See AA.	
**AA2.** IRON	See AA.	See AA.	See AA.	
**AA3.** ZINC	See AA.	See AA.	See AA.	
**AA4.** IODINE	See AA.	See AA.	See AA.	"So when I cut back the salt, I was doing more Himalayan salt was what was in there and became very aware that I was probably going to start running an iodine deficiency."
**AA5.** CALCIUM	See AA.	See AA.	See AA.	*"So choosing dairy products to get to enough calcium. Um, just the bioavailability of calcium and plant-based foods is pretty poor."*
**AA6.** VITAMIN D	See AA.	See AA.	See AA.	

**AA7.** VITAMIN B12	See AA.	See AA. Include reference to other B vitamins as well.	See AA.	
**AA8.** MULTIVITAMIN	See AA.	See AA.	See AA.	
**AA9.** OTHER NUTRIENT OR SUPPLEMENT	If participant mentions a different nutrient that they are concerned about or supplement that they take that is not listed in AA1-AA8.	See AA.<br><br>Includes adopting a plant-based diet for lower cholesterol/saturated fat<br><br>Include comments if a participant notes that they have no nutritional concerns for a plant-based diet.	See AA..	*"I feel like red meat is probably bad for you. Umm… I mean, my cholesterol is okay, but I feel, for cholesterol purposes, I just feel [pause]… it's healthier."*<br><br>*"I take…Magnesium… I also take a probiotic."*
**AA99.** GENERAL REASONS FOR NOT TAKING SUPPLEMENTS	Comments related to not taking vitamins and supplements	Including that they prefer to get nutrients from foods or never thought about it		*"I haven't ever felt the need to take them. I know that the water… I know that a lot… I shouldn't say, no… I have read or been told that a lot of them are not as bioavailable as food sources. And like I don't know… they could degrade in the jar."*<br><br>*"I don't think that [supplements are] necessary. I don't feel deficient in anything. I believe our bodies absorb most of our nutrients through food."*
**BB. SPECIFIC FOODS FOR NUTRIENTS OF CONCERN**	Use to capture discussion regarding practices and ideals related to specific foods that contain nutrients of concern<br>*Use this code when the foods are mentioned, regardless of the context is about how the foods relate to nutrients of concern; also include discussion of why a participant chooses NOT to consume a specific food*			

**BB1.** NUTRITIONAL YEAST	See CC.	See CC.		
**BB2.** WALNUTS	See CC.	See CC. Include discussion about other nuts as well		
**BB3.** SEEDS	See CC.	See CC. Includes chia, hemp and flax.		
**BB4.** ALGAE/SPIRULINA	See CC.	See CC.		
**BB5.** FORTIFIED NON-DAIRY MILKS	See CC.	See CC. Include discussion of non-dairy milks, even if they do not explicitly mention fortification		
**BB6.** SEAWEED	See CC. Includes reference to dulse	See CC.		
**BB7.** IODIZED SALT	See CC.	See CC.		
**BB8.** PROTEIN POWDER	See CC. Include discussion of Collagen Powder.	See CC.		
**BB9.** OTHER FOODS THAT COVER A NUTRIENT OF CONCERN THAT WAS NOT INCLUDED IN THE SURVEY	Comments related to using specific foods to address a nutrient of concern on a plant-based diet that was not included on this list	Examples include: Dairy for calcium or DGLV for iron		*"…if I can get calcium from black beans and from bok choy and other options… cabbage, broccoli all have great calcium in it. I would prefer that."*
**CC. PROTEIN CONSIDERATIONS**		Do not use; parent code.		
**CC1.** PROTEIN SOURCES	Use for general discussion of plant- based protein (includes egg and dairy)	Includes discussion of meeting protein needs or specific protein foods.	Do not use if content is specifically related to other DD.	*"So I guess any kind of legumes and beans… for sure. Nuts are huge and I use them in different ways…And then of*

			categories such a protein complimenting, and sensory factors.	*course, you know, tofu. My kids eat tempeh as well."*<br><br>*"So I know that I can't have a lot of soy because I'm sensitive to it, so that unfortunately rules out… that's part of the reason why I'm so big on beans, right? I have some tofu, but I can't have a lot of it, and I can't do like soy milks or stuff like that just because I react to it."*<br><br>*"Am I getting enough protein? I'm like, you probably are honey, because we all typically get too much protein."*
**CC2.** PROTEIN COMPLIMENTIN G	When participants describe the idea of combining proteins source to create a complete protein			*"I generally follow the guidelines of some kind of grain with bean combination, because it makes it more of a complete protein. I try to incorporate it on some... usually anyways, but try to keep that mindful."*<br><br>*"I read one time that rice and beans makes a complete protein. I don't even know if that's 100% true, but I remembered it and believed it. And so when I have rice I do beans with the rice, almost always. "*
**CC3.** SENSORY FACTORS ASSOCIATED WITH PROTEIN FOODS	Comments describing the importance of sensory factors when choosing protein foods, including taste, texture and satiety			*"But I'm usually looking for something more from a staying power. So looking for protein in a meal as a stick with you."*<br><br>*"I'm not counting macros and trying to make sure everything is correct I'm like… I feel like I would be tired or something if I wasn't getting enough protein."*

DD. MISCELLANEOUS NUTRITION TOPICS		Do not use; parent code.		
**DD1.** IMPORTANCE OF VARIETY/NUTRITIONAL DENSITY TO MEET NEEDS	Comments related to using variety and/or nutritionally dense foods to cover nutritional bases (using variety to cast a wide net)	Include discussion of balance.		*"I think I've just become more aware of seeking out those more higher nutritional, better foods and making sure that even if we don't eat them all the time, that we're still incorporating them into our diet instead of just eating the same things all the time. Maybe that's more of it… is getting more of a variety has been more of a focus for me."*
**DD2.** SOURCE OF NUTRITION INFORMATION	Comments regarding where the participant gets their nutrition information from	This could include health professionals, media, general discussion "I've heard" or "I've read", etc<br><br>Includes using blood work to check levels and inform decision to supplement.	Do not use if general information not specifically related to nutrition; use C1. GENERAL INFORMATION SOURCES in positive deviant codebook	*"So just that everything is grounded in some sort of medical consult with my acupuncturist, or someone in terms of recommendation."*<br><br>*"They have, you know, they have doctors, scientists on the show. And if it would just go over every, every podcast is like a different nutrition topic."*
**DD3.** CHALLENGES TO THE NUTRITIONAL ADEQUACY OF PLANT BASED DIETS	Comments related to experience getting doubted about the nutritional adequacy of plant-based diets	Include questions like "How do you get enough protein, iron, etc.?"		*"What's wrong with you? Where are you getting your protein? You've heard… if you're plant-based, you've heard all the stories….Where are you getting your calcium? All that stuff. And it's just like… we just don't get on the soapbox anymore."*<br>*"What do you eat?" "I can't imagine trying to figure out what to eat if I couldn't eat those things." And also the perception is somehow I'm missing out because I don't eat processed and fast food and meats and things like that."*

**DD4.** PROCESSED FOODS AND HEALTH	Comments that discuss processed foods in the context of health	Include comments related to "junk food vegans" and health of plant-based meat alternatives		*"The more we can get like actual like whole foods instead of just like this processed stuff is like that's really like what's important to base [health around]…And then also that like vegan doesn't equal healthy…."*<br><br>*"I like [plant-based meat alternatives], I enjoy them, but I try not to like, eat them too much because like Yeah, it's just like processed stuff that it's vegan, but it it's not healthy."*
**DD5.** FOOD HACKS	Creative ideas for integrating foods with nutrients of concern to make a plant-based diet easier	Include examples of using plant-based alternatives to make a food that is not normally plant-based plant-based (i.e., cashew cheese); Can include ingredients that are not specifically mentioned in **CC. SPECIFIC FOODS FOR NUTRIENTS OF CONCERN**		*"For the almond milk and I'd say what we incorporate them in, we do we make vegan pancakes so that calls for a plant milk."*<br><br>*"I dry and powder greens, and then I put that in everything. So to try to keep the calcium because there's a lot of calcium in dark greens, so that's one of them."*<br><br>*"Oh, flax. Yeah and I use that as just egg substitute."*<br><br>*"All my carrot tops get turned into pesto. And Kale stem pesto and such….And if I can't process it, toss it into the freezer so that I can deal with it at another time."*<br><br>*"It's called the, it's the vegan feta pasta…. it uses cashews, nutritional yeast, garlic powder, pasta and tofu. And basically you*

				kind of just, you grind it up into like uh, it's like, it's like a vegan feta."
**DD6.** OTHER CONCEPTS RELATED TO NUTRITIONAL ASPECTS OF A PLANT-BASED DIET.	Capture other concepts related to nutrition or nutritional planning on a plant-based diet not captured elsewhere in the codebook.	Include comments related to the GI adjustment needed for plant-based diets due to increased fiber<br><br>Include comments that show a general understanding of basic nutritional concepts not tied to a specific nutrient or supplement, including but not limited to bioavailability, nutrient needs, needs for nutritional planning on a plant-based diet<br><br>Include comments related to changed in nutritional needs throughout the lifecycle (i.e. aging, etc)		*"…they need to figure out which proteins they can digest best and enjoy best. Whether it's beans, whether it's tofu, whether it's like eating just a ton of your protein, heavier vegetables. I know some people are really bothered when they're adjusting to eating more beans."*<br><br>*"There were definitely was like uh a period where consuming the increased amount of fiber, it… Yeah, there's like an adjustment period with your thought to get used to it."*<br><br>*"I've gotten to the point where I tell people the only things about nutrition that I know to be completely true are water is good for you in moderation, and vegetables are probably good for you."*<br><br>*"And obviously there's a whole other side of that, that I know about is that, you know, your body only takes up a certain amount of, you know, the bio availability side of it that your body, like, digests a certain amount."*<br><br>*"And we evolved with the plants, eating the plants and eating a huge variety of plants, far above what people today eat….And I don't think that our physical bodies have evolved with the pace of the*

				changes in food that we've introduced and become dependent on as a species. So I think it's essential for… to continue our physical wellbeing. The nutritional deficiencies are creating long term changes…"
